# Ambroise Pare and His Times, 1510—1590

**Published:** 1898-09

**Authors:** 


### Reviews of Books.

**Ambroise Paré and his Times, 1510—1590.** By STEPHEN PAGET. Pp. xiv., 309. New York: G. P. Putnam's Sons. 1897.

A more interesting personality than that of the subject of this memoir can hardly be found in the whole history of medicine, and we owe a debt of gratitude to Mr. Stephen Paget for giving us in such a readable and delightful form a biography of this remarkable French surgeon.

He lived a long life, dying at the age of eighty years, "a learned man, and the chief of all surgeons; who, even against the times, all his life talked and spoke openly for peace and for the people; which made him as much beloved by the good as he was begrudged and hated by the wicked." So we read in the memoirs of Pierre de L'Estoile, from which Mr. Paget quotes freely. When we know that the life of Ambroise Paré extended from Louis XII. to Henri IV., from Maximilian to Philip of Spain, from Henry VIII. to Elizabeth; and that among his contemporaries were Ignatius Loyola and Saint Theresa, Luther and Erasmus, Calvin and Knox, Shakspere and Rabelais, Raphael and Titian, Paracelsus, Servetus, Sylvius, and Vesalius, we can realize the stirring times in which he lived. He followed the wars, off and on, for thirty-two years, and thus gained a most valuable experience in the treatment of wounds which he was not slow in turning to account. At first he followed the directions he had received from his predecessors, who considered that wounds made by firearms were venomous by reason of the gunpowder and needed to be cauterised with scalding oil; but he soon found that wounds thus treated became inflamed and swollen, whereas those treated by "a digestive made of the yolks of eggs, oil of roses, and turpentine" healed without any inflammation, so he resolved never more to burn thus cruelly poor men with gunshot wounds. He practised in Paris for more than half a century, and was surgeon to four kings. He made no pretensions to scholarship and never learned Greek or Latin. He loved the practice of his profession, and did more for the "art" of surgery than almost any of his predecessors or contemporaries. He is best known to posterity by his being the first to use the ligature for arresting hemorrhage after amputations. Before his time the actual cautery was always used for this purpose; but he was so struck with the diabolical cruelty of this plan, that he adopted the ligature which he had often used to vessels bleeding in an ordinary wound and which was as old as Galen. When it came to his thoughts that he might use the ligature in amputation-wounds, he conferred with Estienne de La Rivière and other Paris surgeons, and they agreed together to make trial of it, having the cauteries ready to hand in case the ligature should fail.

If we had space we should like to quote freely from Mr. Paget's book, which is full of delightful gossip of the French kings and their courts and the wars in which they indulged.

During his travels Ambroise Paré kept a diary, which is here reproduced under the quaint heading "Journeys in Diverse Places;" it is full of the amusing and shrewd observations of this philosopher as well as surgeon. The book is profusely and well illustrated, and does honour both to author and publisher.

